# A review of the serotonin transporter and prenatal cortisol in the development of autism spectrum disorders

**DOI:** 10.1186/2040-2392-4-37

**Published:** 2013-10-08

**Authors:** Roselyn Rose’Meyer

**Affiliations:** 1 School of Medical Sciences, Griffith University, Gold Coast Campus, Parklands Drive, Southport, Queensland 4222, Australia

**Keywords:** Autism spectrum disorder, Cortisol, Serotonin, SERT

## Abstract

The diagnosis of autism spectrum disorder (ASD) during early childhood has a profound effect not only on young children but on their families. Aside from the physical and behavioural issues that need to be dealt with, there are significant emotional and financial costs associated with living with someone diagnosed with ASD. Understanding how autism occurs will assist in preparing families to deal with ASD, if not preventing or lessening its occurrence.

Serotonin plays a vital role in the development of the brain during the prenatal and postnatal periods, yet very little is known about the serotonergic systems that affect children with ASD. This review seeks to provide an understanding of the biochemistry and physiological actions of serotonin and its termination of action through the serotonin reuptake transporter (SERT). Epidemiological studies investigating prenatal conditions that can increase the risk of ASD describe a number of factors which elevate plasma cortisol levels causing such symptoms during pregnancy such as hypertension, gestational diabetes and depression. Because cortisol plays an important role in driving dysregulation of serotonergic signalling through elevating SERT production in the developing brain, it is also necessary to investigate the physiological functions of cortisol, its action during gestation and metabolic syndromes.

## Review

### Definition of autism spectrum disorder

Autism spectrum disorder (ASD) is a neurodevelopmental disorder presenting in the first 3 years of life that is strongly correlated with changes in neural growth during prenatal and post natal periods [1]. A child with ASD presents with the following: defects in social interaction and communication [2]; repetitive stereotypic behaviour or movements [2]; deficits in language acquisition [3], failure to assume meaning from normal social cues and a fixation on a maintained uniformity of routine [4]. Furthermore, this disorder is also associated with sensory abnormalities with a low threshold to sensory inputs that result in avoidance behaviours [5]. ASD is generally viewed as a male predominant disorder with a ratio of 4:1 to females [6].

Autism is classified as a pervasive developmental disorder and clinicians and researchers use the term ASD to include autism, Asperger’s syndrome and pervasive developmental disorder not otherwise specified (PDD-NOS) (for review of symptoms see [2]). The primary symptoms of ASD have previously been defined in the *Diagnostic and Statistical Manual of Mental Disorders, 4th Edition* (DSM-IV, American Psychiatric Association) with some impairments controversially reclassified in the recently released DSM-V [7].

Over the past 20 years there has been an increase in the frequency of the disorder with present rates of ASD being diagnosed at about 2% for children in the USA [8]. A number of reasons have been advanced for this increase, including changes in administrative classifications, policy and practice changes and increased awareness [2]. It is believed that surveillance and screening strategies for early identification of ASD could allow early treatment and improved outcomes.

Nevertheless, hereditary and environmental factors that occur during gestation and postnatal periods leading to significant aberrations in neural organisation and cortical network development are the most likely causes of ASD [9].

### Diagnosis of autism spectrum disorder

It has been difficult to diagnose ASD until the affected child is 3 years of age, as the clinical signs are not easily identifiable and language development is delayed [2]. Some families report social deficits (such as facial expressions, non-verbal gestures and reduced interaction) within the first few months after birth [10]. A retrospective video study published by Baranek in 1999 [11] observed the symptoms of ASD in children at 9 to 12 months and has suggested a number of early intervention procedures that involve assessment of sensory processing and sensory motor functions in addition to recording social responsiveness during infancy to diagnose ASD. Studies investigating infants at risk (siblings of affected children) have reported that deficits in communication and social interactions can be identified as early as 6 months of age [12,13]. These observations indicate that relevant neurochemical events that alter neuroanatomical growth occur early in the development of the CNS (central nervous system) [14]. Therefore, it is suggested that if we can understand the combination of events that cause the development of ASD, then we can identify women and children at risk and conceivably develop treatments to reduce symptoms associated with this condition.

### Aetiology of ASD

A combination of clinical, neuroimaging, neuropathological and neurochemical studies of individuals diagnosed with ASD have reported disorders in the neuronal cortical organisation causing deficits in information processing in the nervous system [15]. This includes alterations in neural synaptic and dendritic organisation that modifies brain structure, coupled with abnormal patterns of brain growth during the early years in brain regions involved in the development of social, communication and motor abilities [9].

ASD has been shown to be a highly genetic disorder. Heritability estimates from family and twin studies suggest that about 90% of variance is attributable to genetic factors [16], with 60 to 92% of monozygotic twins concordant for ASD (depending on the symptoms) compared to 10% of dizygotic twins [17]. The risk of developing ASD has recently been estimated to be 18.7% for siblings of individuals diagnosed with ASD. This reoccurrence risk is higher than previously reported rates of 3 to 10%, once data which have been distorted due to families who stop having children once a child has been diagnosed with the developmental disorder have been removed [18].

ASD is recognised as a multifactorial disorder with other risk factors contributing to the phenotype. Studies have shown that serotonin and genetic differences in serotonin transport could contribute to the development of ASD, as serotonin has a vital role in stimulating cell proliferation in the developing brain during pre- and postnatal periods as well as in early childhood [14]. Studies have shown macroscopic and microscopic brain abnormalities may occur in utero and that the pathogenesis of this condition may begin during the prenatal period [15,19,20].

### Comorbid conditions and ASD

Comorbid conditions are common in children and families with ASD. Parents of affected children have increased rates of stress, anxiety and depression [21]. Comorbid behavioural and developmental disorders in people diagnosed with ASD include intellectual delays, inattention, attention-deficit hyperactivity disorder, aggression and disruption, depression or anxiety, sleep disruption or sensory differences [22,23]. Other comorbidities include gastroesophageal reflux, flood selectivity and neurological disorders, such as tics, seizures or migraine [24]. Many of these conditions have been linked to the dysregulation of serotonergic systems [25-34]. Abnormalities in serotonergic function have been linked with ASD since Schain and Freedman reported hyperserotonemia in 1961 [35]. This observation has been confirmed in subsequent studies where in 25 to 33% of individuals diagnosed with ASD, whole blood serotonin levels were found to be elevated [36]. Furthermore, family members of children diagnosed with ASD with hyperserotonemia also exhibit raised blood serotonin levels [37].

### Physiological actions of serotonin and its regulation by serotonin reuptake transporter (SERT)

Serotonin is synthesised from the essential amino acid L-tryptophan. L-tryptophan is hydroxylated to 5-hydroxytryptophan (5-HTP) then decarboxylated by aromatic-L-amino acid decarboxylase to serotonin [38]. Serotonin produced peripherally does not cross the blood-brain barrier of the mature brain and as such, the neurotransmitter has to be synthesized in the CNS. It must be noted, however, that the blood brain barrier is not fully functional in the developing human brain until 2 years of age and it has been hypothesized that elevated platelet serotonin may alter the monamine levels during early brain development in autistic children [39]. After its release from serotonergic neurons, serotonin will bind its receptors at the synaptic site to activate intracellular signalling pathways to induce physiological effects. The actions of serotonin are then terminated when it is rapidly taken up by a SERT. Serotonin is catabolised by the mitochondrial enzyme monoamine oxidase A to its metabolite 5-hydroxyindolacetic acid (5-HIAA).

The behavioural effects of serotonin are numerous as it regulates mood, appetite, body temperature, arousal, moderates pain sensitivity, sexual behaviour and hormone release [14]. Other than its actions as a neurotransmitter in the CNS, serotonin is released in the periphery to mediate a range of physiological activities. The SERT proteins are localised to the presynaptic terminals of the serotonergic neurons [40] and is found in limited places such as the specialised cells of the gut [41], placenta [42], lung [43], pancreas [44] and adrenal chromaffin cells [45], blood lymphocytes [41] and platelets [46]. In the periphery, tissues take up and store serotonin in vesicles where it is released in response to local stimuli [47]. The importance of SERT in regulating normal tissue serotonin levels was demonstrated in SERT-deficient mice, where platelets and most peripheral organs were found to be empty of serotonin. This work showed that there are no compensatory mechanisms such as other monoamine transport systems that could re-establish tissue serotonin levels [47]. Platelets express SERT proteins that are identical to brain SERT [48] and they acquire serotonin released by the enterochromaffin cells as they circulate through the gut. Serotonin captured by platelets have a role in injury where serotonin release can alter blood flow [49] as well as stimulating the production of adhesive alpha-granular proteins in activated platelets [50], and as indicated previously, abnormal levels of platelet serotonin have been observed in ASD-diagnosed children and their relatives. Chronic use of selective serotonin reuptake inhibitors (SSRIs) decrease platelet serotonin content, protects against myocardial infarction or intensifies bleeding episodes [51,52]. Subsequently, excess serotonin uptake may contribute to platelet hyperactivity and thrombosis as reported, with an association of SERT polymorphisms with cardiovascular disease [53]. The presence of SERT proteins on the placental brush border membrane, and the role of serotonin in vascular function indicates that serotonin may have a role in placental function and therefore, growth and development of the human foetus [42]. Serotonin is synthesized by enteric neurons and enterochromaffin cells of the gut where it locally regulates digestive processes [54,55]. It can be released into the blood or into the lumen of the gut where it can modify intestinal transport, proliferation of gastrointestinal epithelium and modulation of intestinal motility. Serotonin inhibits gastric acid secretion and may be an endogenous enterogastrone, and stimulates the production and release of gastric and colonic mucus [56]. In the gastrointestinal tract, serotonin acts as a crucial signalling molecule initiating responses such as nausea, vomiting, and peristaltic and secretory reflexes [57]. Serotonin is taken up into the β-cells of the pancreas, where it is stored in granules that contain insulin to modify insulin release [44].

Serotonin has specific functions in the CNS and in the periphery where it regulates many physiological activities. Problems in serotonergic signalling in some of these systems have been implicated as comorbidities that occur with ASD as discussed in the previous section.

### The role of serotonin in neuronal function and development

An appreciation of the roles of serotonin as a neurotransmitter and in neuronal growth, particularly during early development, reveals why perturbations in the serotonin signalling systems could contribute to the development of ASD. The serotonergic system is one of the most widely distributed and one of the earliest to develop in the mammalian embryo [58].

In the brain, the majority of serotonergic neurons are located in the median and dorsal raphe nuclei, which project to the cortex or hippocampus, respectively. The cell bodies of the serotonergic neurons are found in clusters, most of which are located in the raphe nuclei of the midbrain, pons and medulla [59]. The serotonergic system innervates virtually all areas of the brain and serotonergic neurons can be detected in the human brain from the fifth gestational week, where they grow and rapidly multiply [60].

With regards to the control of behaviour the two most important clusters of serotonergic neurons are found in the dorsal and medial raphe nuclei, both of which send neuronal projections to the cerebral cortex. As well as its role as a neurotransmitter, serotonin acts as a trophic or differentiation factor in early neurogenesis, where changes in serotonin levels during brain development have been reported to alter neuronal differentiation [61,62].

SERT is a significant contributor to moderating neuronal serotonin levels. There are a significant number of internal and external influences that can alter SERT expression and function from early embryonic stages through to adolescence. And although these influences continue to guide SERT expression and activity throughout adulthood, SERT activity during early periods of human development appear to be vital for guaranteeing normal development [63]. In rodent studies, decreased or increased brain serotonin during the postnatal period of development results in the disruption of synaptic connectivity in sensory cortices in the brain [64-66]. In human studies, serotonin has been demonstrated to be important for prenatal and postnatal brain development [67]. Irregularities in brain serotonin levels can cause asymmetric development of the serotonergic system which leads to incorrectly connecting neural circuits [68]. Changes in serotonergic function and signalling have been found to be associated with ASD [67]. Humans undergo a period of high brain-serotonin synthesis capacity during childhood, a process affected in autistic children [69]. In humans, serotonin activity as measured by the cerebrospinal fluid (CSF) levels of the metabolite 5-HIAA, is higher in children when compared to adults [70,71]. 5HIAA levels in the CSF of children with ASD has been reported to provide a reliable measurement of neural serotonin turnover [14]; however, many studies have indicated a reduction or no change in CSF 5HIAA levels in individuals diagnosed with autistic disorders [72,73]. As such, the levels of serotonin metabolites present in the CSF of ASD-diagnosed individuals is still not firmly established, nor is the impact of blood serotonin levels and how they relate to brain serotonin levels [68].

Functional neuro-imaging studies using positron emission tomography (PET) have shown diminished serotonin synthesis in children with ASD between the age of 2 and 5 years [69]. The short-term depletion of L-tryptophan has been shown to exacerbate repetitive behaviour and elevate anxiety in autistic individuals [74], and drug treatment with selective serotonin re-uptake inhibitors, which interact with SERT, have been shown to be effective in decreasing repetitive and/or obsessive behaviour in some but not all autistic individuals [75].

The main issue with the studies investigating 5HIAA levels in the CSF, platelet serotonin or neuronal serotonin synthesis is that they are completed in cohorts of individuals diagnosed with ASD at different ages and growth stages. The role of serotonin in neuronal development changes throughout infancy and childhood as do the environmental factors that can alter serotonergic systems and function.

### SERT structure and function

Intracellular and extracellular serotonin levels are controlled through tissue SERT expression levels and transporter activity. SERT is a protein consisting of 630 amino acids and has a similar structure to the noradrenaline transporter (NET) and dopamine transporter (DAT). *In vitro* experiments with SERT have demonstrated that phosphorylation state of the transporters is controlled by several kinase and phosphatase signalling pathways which alters movement of serotonin through the transporter [76]. SERT-medicated serotonin uptake is driven by a Na^+^/Cl^-^ transmembrane ion gradient [77]. SERT proteins can be regulated by numerous protein kinase (PK) linked pathways, which include the signalling molecules protein kinase C (PKC), protein kinase G (PKG) and p38 mitogen-activated protein kinase (MAPK) [78-80]. Extracellular serotonin can induce the phosphorylation and downregulation of SERT through PKC signalling pathways [81]. Several G-protein coupled receptors such as adenosine, histamine and α_2_-adrenergic receptors also modulate SERT activity [79,82] as well as inflammatory cytokines such as IL-10 and IFN-γ, and TNF-α [83-85]. SERT is the major mechanism by which serotonin uptake from extracellular fluid occurs; however, when SERT function or expression is altered and the levels of serotonin are elevated, other monoamine transporters that have a lower affinity for serotonin such as DAT and NET will transport serotonin [86].

Gain-of-function SERT-coding variants have been reported with some SNPs in the gene that encodes SERT, SLC6A4, causing a change in the amino acid sequence of SERT [87]. These are rare genetic variants, which represent a frequency of much less that 1% of the population. Human variants such as Ile425Leu, Phe465Leu and Leu550Val when expressed in HeLa cells have been shown to exhibit a gain of serotonin transport phenotype due to elevated expression of the transporter and altered regulation via the PKG/p38 MAPK signalling pathways [88] whereas the Gly56Ala variant increases in serotonin transport across cell membranes with no changes in transporter numbers [89].

The Gly56Ala variant has been reported to have a higher prevalence in individuals diagnosed with ASD and is associated with both sensory aversion and rigid-compulsive behaviour [90], whereas the Ile425Val variant (albeit the same locus, different amino acid substitution to Ile425Leu) is associated with obsessive-compulsive disorder and Aspergers syndrome [91].

To further investigate the gain of function activity of the Gly56Ala variant *in vivo*, transgenic mice expressing the SERT Ala56 were developed and exhibited normal growth patterns and fertility [92]. Further studies on the transgenic Gly56Ala mice showed they had increased CNS serotonin clearance, enhanced serotonin receptor sensitivity and hyperserotonemia. The mice also exhibited alterations in social function, communication and repetitive behaviours [93]. Similarly in other transgenic mice, overexpression of the SERT protein caused a reduction in the brain region levels of serotonin and enhanced sensitivity of postsynaptic 5-HT_2A_ receptors was observed [94].

In summary, SERT function is regulated via several signalling systems. When SERT tissue expression increases or SERT function is enhanced, serotonin uptake is increased, which diminishes levels of the neurotransmitter in the synaptic cleft and causes an increase in sensitivity of postsynaptic serotonin receptors to serotonin. In human and animal studies where increased SERT function was observed neurological symptoms similar to those observed in ASD were reported.

### Genetics of SERT in ASD

ASD is a genetically inherited disorder and SERT has been the focus of much research due to its prominent role in serotonin homeostasis. SERT is encoded by the *SLC6A4* (Solute carrier family 6 (neurotransmitter transporter, serotonin, member 4) gene. Several gene variants of *SLC6A4* that are associated with ASD alter the structure, function or expression of SERT [47]. SERT functions in all serotonergic systems through transport-mediated regulation of serotonin release and activation of homo- and hetero-receptors in brain, platelets and peripheral organs. Changes in the function of SERT alters the affinity and expression of serotonin receptors, as well as the pharmacokinetics of serotonin [47]. Human *SLC6A4* maps onto chromosome 17q11.2 [95] and it is one of several genetic loci that has been identified as predisposing to ASD [96,97]. The gene is composed of 15 exons spanning approximately 40 kb. The sequence of the transcript predicts a protein containing 630 amino acids with 12 transmembrane domains. Alternative promoters and splicing of the code involving exons 1A, B, C and the 3’ untranslated region results in variable mRNA products. Polymorphisms, which alter the expression of the *SLC6A4* gene and therefore, SERT protein levels, have been the focus of research into the genetic inheritance of ASD and as such, polymorphisms within the 5-HTTLPR promoter sequence, mutations in the coding sequence, or intronic mutations of the SERT have been reported to be linked to ASD in some but not all studies [1,47,89]. Two major polymorphisms in the *SERT* gene have been of major interest. First, a variable number of tandem repeats (VNTR) occurs in the second intron of the gene [98]. Second, basal and induced human *SERT* gene transcription is differentially modulated by allelic variants of the *SERT* gene promoter [98]. The SLC6A4 5-HTTLPR promoter sequence is located approximately 1 kb upstream of the transcription initiation site, contains two variable repeat length polymorphisms known as long (L) with 16 repeat elements, or the 44-base pair (bp) shorter (S) variant with 14 repeat elements [95,99], which determines the expression of the SERT in the pre-synaptic axonic membranes. The L/L variant of the 5-HTTLPR promoter region expresses significantly (1.4- to 2.0-fold) more transporter protein compared to L/S or S/S variants [100,101]. Higher SERT mRNA levels and increased serotonin uptake is evident in lymphoblasts of the L/L homozygotes compared to those with at least one copy of the S allele [102]. The S/S polymorphism reduces transcriptional efficiency of the SERT promoter to reduce the SERT expression and serotonin uptake in lymphoblasts [102]. Therefore the L/L variant was proposed to contribute to a lower concentration in the synaptic cleft, although this is not consistently supported by the literature [100]. The S/S polymorphism has been reported to be associated with psychiatric disorders including neuroticism [99] schizophrenia [103], anxiety [104], depression [105], suicide [106,107], ASD [108] and GI syndromes such as irritable bowel syndrome [109,110].

The L variant is also correlated with higher rates of serotonin uptake into platelets [111,112], suggesting that the S variant may act as a dominant allele [101]. The results of multiple studies of the 5-HTTLPR and ASD have been inconsistent, showing an association of the short [113-116] or long allele [117,118], and some studies have found no association [119-121].

There is substantial evidence that ASD is genetically inherited and current research has evaluated many polymorphisms that could alter SERT expression in ASD without consistent results, suggesting that the genetic changes associated with SERT have yet to be identified. At the same time ASD is associated with multiple polymorphisms in the *SLC6A4* gene with individuals heterozygous for the Gly56Ala plus 5-HTTLPR L/L promoter variants [73], and other studies into *SLC6A4* and other psychiatric disorders suggests that haplotypes (multiple alleles) that include the 5-HTTLPR variants may contribute to this disorder [122,123].

### Prenatal conditions and ASD

Specific maternal illnesses, conditions and treatments can result in adverse neurodevelopmental outcomes in children [124]. Perinatal complications place an infant at significant risk for mental, neurological and behavioural disorders [125]. Maternal metabolic conditions may increase the risk of ASD. Maternal Type 2 diabetes, hypertension, and obesity have been identified as risk factors for ASD and other developmental disorders [124,126]. Prenatal factors such as advanced maternal (and paternal) age, bleeding or gestational diabetes have been associated with the risk of ASD [127,128]. An Australian study has linked an increased risk for the development of intellectual disabilities (which included ASD) with maternal asthma [124]. Another report observed that maternal asthma increased the risk of adverse fetal and maternal outcomes such as low birth weight, preeclampsia, hypertensive disorders and gestational diabetes [129]. A high rate of autoimmune diseases occurs in families with ASD indicating that immune dysfunction could combine with other environmental factors in the development of ASD [130]. Parental psychiatric history and prenatal environmental factors also contribute to an increased risk of developing ASD [131].

Prescriptions taken during the pregnancy, length of labour, viral infections, abnormal presentation during birth, and a low birth weight could also be factors that predict outcomes of infantile ASD [125]. Furthermore, the risk of ASD development in preterm babies who are small for their gestational weight is increased, whereas preterm babies who are large for their gestational weight have a reduced risk of ASD [132]. Foetal stress during delivery may also increase the risk of ASD [128]. Overall, epidemiological studies have identified factors including gestational diabetes, stress, infections and inflammatory disorders as prenatal risk factors for ASD. Many of these conditions are known to directly or indirectly elevate cortisol levels [133]. Taking oral corticosteroids during pregnancy confers increased risk of lower birth weight and congenital malformations [129]. Elevated prenatal cortisol is known to negatively affect the behaviour of newborn children with increased irritability, attention and temperament problems [134,135]. Excess plasma cortisol levels have been implicated in the aetiology of comorbid illnesses associated with ASD, such as depression, anxiety, dyspepsia and migraine [136,137] (see Table 1). Furthermore, elevations in plasma cortisol and platelet serotonin levels have been observed in schizophrenic patients [138]. Hence, there is evidence to indicate that excess cortisol levels co-exist with serotonin-selective pathologies.

**Table 1:** **Clinical outcomes of tissue-specific glucocorticoid excess **[139-142]

**Tissue/system**	**Symptom**
Nervous system	Anxiety
	Insomnia
	Depression
	Memory dysfunction
Liver	Gluconeogenesis
	Lipogenesis
Skeletal muscle	Insulin resistance
	Atrophy
	Fatigue
Bone	Osteoporosis
Blood	Elevated blood glucose
	Impaired fasting glucose
	Hypokalaemia
	Dyslipidaemia
Adipose tissue	Obesity
	Fat redistribution
	Weight gain
Cardiovascular system	Hypertension
	Pre-eclampsia
	Sodium/water retention
	Oedema
Immune system	Immune suppression - infections
	Delayed wound healing
Skin	Hirsuitism
	Striae
	Bruising
	Thinning
	Acne
	Delayed wound healing
Other	Dyspepsia
	Increased appetite
	Impaired growth in children
	Suppressed HPA-axis function
	Cataracts
	Glaucoma

### Cortisol regulation

Cortisol levels rise significantly during gestation and cortisol is an important hormone involved in the development of the fetus. The following sections will review cortisol regulation, physiological actions and its role in metabolic syndromes. The modulation of cortisol by the reproductive hormones and its contribution to gestational diabetes will also be discussed. Glucocorticoids such as cortisol are steroid hormones that are released by the adrenal cortex to regulate carbohydrate metabolism. The hypothalamic release of corticotrophin releasing hormone (CRH) regulates the secretion of adrenocorticotrophic hormone (ACTH) from the anterior pituitary gland, which in turn stimulates the release of cortisol from the adrenal gland. It is a tightly regulated system in which increased plasma cortisol leads to feedback inhibition of both CRH and ACTH [143]. Normal plasma levels of cortisol vary significantly according to the time of day with levels highest in the morning at 0800 h (138 to 635 mmol/L); at 1600 h the cortisol level is 83 to 413 mmol/L, and at 2000 h it is 50% of the 0800-h level. Urinary cortisol (24-h urine) in children is 5.5 to 74.0 mmol/L and in adolescents it is 14.0 to 152.0 mmol/L [139]. The human hypothalamic pituitary adrenal axis (HPA) refers to the hormonal feedback mechanisms that regulate cortisol levels in the body. The HPA is controlled with circadian rhythms [144] and ultradian rhythms with discrete pulses of ACTH and glucocorticoids to regulate cortisol levels in the body [144,145].

As part of the stress responses many brain regions, including the limbic and sympathetic systems, regulate HPA activity [146,147]. Serotonin can regulate the HPA axis through serotonergic pathways linked to the hypothalamus or hippocampus to stimulate CRH or ACTH release or acting as a local paracrine factor in the regulation of cortisol from the adrenal cortex [148,149]. HPA axis dysregulation is caused in part by the release of various inflammatory cytokines, including TNF-α, IL-1 and IL-6 where they stimulate CRH production [148].

Cortisol can be inactivated by the enzyme 11β-hydroxysteroid dehydrogenase (11β-HSD)-2, which is found in mineralocorticoid receptor-rich tissues such as the kidney [150] and adipose tissue [151] but not the liver. Cortisol has a high affinity for the mineralocorticoid receptor, however, inactivation to cortisone by 11β-HSD2 renders it unable to bind to the mineralocorticoid receptor and facilitates aldosterone binding to this receptor [152]. In tissues, including liver, adipose, brain [150] and blood [153], cortisol can be regenerated from its inert form, cortisone, by the enzyme 11β-HSD-1 [154]. 11β-HSD1 is a reduced nicotinamide adenine dinucleotide phosphate (NADP(H))-dependent microsomal enzyme that converts cortisone into cortisol. Expression of 11β-HSD1 can also be induced in many other tissues including fibroblasts, skeletal and smooth muscle, and immune cells [155-158]. The 11β-HSD1 enzymes are known important regulators of hormone action at the tissue level [150]. Adipocyte-derived leptin and oestrogen upregulate 11β-HSD1 [159,160] to increase both intracellular and circulating cortisol levels. In the circulation, over 90% of cortisol is bound to corticosteroid binding globulin (CBG). It is only the unbound free portion that is able to diffuse into the cell and exert its effects.

Glucocorticoids are abundant highly widespread nuclear hormones [161,162]. They exert their actions in almost all tissues, influencing the expression of a large proportion of the human genome. Binding of the glucocorticoid to its receptor changes the transcription rates of target genes. A large number of molecules participate directly or indirectly in the signalling cascade [161,163]. Glucocorticoids are pivotal in regulating many aspects of resting and stress-related homeostasis. They are released as part of the stress response and have a catabolic effect to liberate substrates for mitochondrial oxidation [164].

### Physiological effects of cortisol

Cortisol stimulates gluconeogenesis and fatty-acid mobilisation in the liver and adipose tissue. One of the most important effects of cortisol is that it upregulates glucose production. In the liver, cortisol increases the expression of the gluconeogenic enzymes phosphoenolpyruvate carboxykinase-C (PEPCK-C) and glucose-6-phosphatase (G6Pase), which releases glucose from glycogen into the circulation [165]. Glucocorticoids primarily act by the activation of the glucocorticoid receptor (GR) and the regulation of transcription. The GR is a ligand-regulated nuclear receptor that belongs to the steroid hormone receptor family. The GRs are expressed in almost all tissues. Upon binding by cortisol, the GR moves to the nucleus, binds specific glucocorticoid response elements (GRE) and recruits co-activators and co-repressors, which can increase or decrease gene transcription [166,167]. The GR can also alter nuclear translation without the GRE [168] and cortisol can also exert non-genomic actions in stimulating endothelial nitric oxide production [169].

### Cortisol and metabolic syndrome

Prolonged elevation of cortisol can lead to hyperglycaemia and insulin resistance as observed in Type 2 diabetes and metabolic syndrome [164,170,171]. Glucocorticoids directly inhibit insulin release from the β pancreatic cells [172,173]. Cushing’s disease or hypercortisolism is a hormonal disorder that causes elevated cortisol levels. Adverse effects linked to upregulation of the HPA axis occur in patients with Cushing’s syndrome, causing outcomes such as osteoporosis, immunosuppression, hypertension, sleep disorders and glucose intolerance [174]. Other effects observed in women with high levels of cortisol include muscle wasting, striae, hirsuitism, acne, menstrual abnormalities and infertility [140]. Individuals who develop pathological states of glucocorticoid excess exhibit all the features of metabolic syndrome, however, Cushing’s syndrome is rare and the circulating levels of cortisol are normal in the majority of patients with obesity and Type 2 diabetes. One hypothesis is that tissue-specific deregulation of cortisol metabolism may be involved in the intricate pathophysiology of metabolic syndrome [175] and that changes in the expression of 11β-HSD1 increase tissue-specific levels of glucocorticoids [176,177]. Inhibitors of 11β-HSD1 are being evaluated in clinical trials for the treatment of Type 2 diabetes. The drugs work by decreasing the amount of cortisol generated in the liver and adipose tissue, thereby reducing gluconeogenesis and fatty-acid breakdown [178]. Ketoconazole is a steroid synthesis inhibitor that lowers cortisol in Cushing’s disease [179] by reducing plasma cortisol levels without affecting the CRH secretion in healthy adults [180].

### Cortisol levels during pregnancy

Circulating and bound levels of cortisol both increase as gestation proceeds to levels that are similar to those detected in Cushing’s syndrome, with plasma levels of cortisol reaching 2- to 3-fold higher than observed in non-pregnant women [181-184]. The rise in cortisol levels begins in week 11 and continues to rise and peak between the first and second trimester to a maintenance level during the third trimester [185]. The salivary cortisol in pregnant women is twice as high as in non-pregnant women in the third trimester [181,186], and the circadian rhythm of cortisol is partly blunted [181,186]. Plasma ACTH levels rise throughout pregnancy reaching a peak during labour and delivery, with placental ACTH production being a significant contributor to hypercortisolism in pregnancy [181]. CRH is synthesized in the human syncytiotrophoblast and released into both the maternal and fetal blood in significant quantities [187,188]. In contrast to the hypothalamic CRH system, the placental production of CRH is stimulated by glucocorticoids [189], providing a positive feedback system, which is a unique characteristic of placental CRH and indicates a role for CRH in late stages of gestation [190]. Exposure to elevated cortisol levels early in the pregnancy may accelerate placental synthesis and the release of corticotrophic releasing hormone to precipitate early delivery [191]. Placental hypersecretion of CRH mid gestation has been proposed as a predictive marker of subsequent preterm delivery [192]. It has been postulated that towards the end of the gestation period CRH stimulates cortisol production in the foetal adrenal glands [193,194].

### The effect of reproductive hormones on cortisol levels during pregnancy

At the onset of gestation, progesterone and oestrogen are secreted by the corpus luteum in moderate amounts. The placenta then takes over progesterone and oestrogen synthesis for the rest of the pregnancy. Progesterone secretion can increase up to 40-fold by the third trimester during a normal pregnancy and oestrogens levels increase up to 30-fold by full term. Both progesterone and oestrogen receptors are expressed in the pancreatic islets of Langerhans and regulate β-cell viability and function [195]. Progesterone has a faster association for CBG than cortisol and higher levels of progesterone during pregnancy may displace cortisol from CBG, increasing plasma cortisol [196]. Progesterone can also be converted to cortisol via 17-α-hydroxylase and 21-hydroxylase in the adrenal glands [197].

Increasing placental oestrogen stimulates the production of CBG by the liver, therefore altering the pharmacokinetics of cortisol [181]. Fetal ACTH secretion is increased as oestrogen is secreted in increasing amounts by the placenta in late gestation [198]. Oestrogen escalates and androgens reduce basal and stimulated ACTH secretion [199]. The levels of circulating ACTH and cortisol concentrations change during the normal menstrual cycle in women, where the highest concentrations of these hormones are measured in tandem with the highest circulating levels of oestrogen [200]. Therefore, oestrogen levels rise in women either during the menstrual cycle or pregnancy, altering CBG production, upregulating 11β-HSD1 levels or directly stimulating the pituitary gland, causing an overall rise in plasma cortisol.

### Gestational diabetes

One of the prenatal risk factors for ASD is gestational diabetes. During late pregnancy mothers can develop insulin resistance [201]. Gestational diabetes occurs in 2 to 3% of all pregnant women [202], although more current estimations indicate up to 14% of all pregnancies are affected by gestational diabetes depending on the test criteria used [203]. Elevated cortisol levels have been measured in pregnant women with impaired glucose tolerance or gestational diabetes [204]. Gestational diabetes is a growing health concern for both the short- and long-term outcomes for both mothers and their offspring [205]. Glucose tolerance deteriorates in all women where a diminished peripheral sensitivity to insulin develops [206]. Normal pregnancy especially the third trimester is characterised by elevated metabolic stress on maternal lipids and glucose homeostasis, which includes insulin resistance and hyperinsulinemia [207,208]. Progesterone receptors expressed in pancreatic islet cells inhibit β-cell proliferation to reduce insulin secretion and glucose tolerance during pregnancy [209]. Known risk factors for gestational diabetes include excessive weight, advanced maternal age, family history of Type 2 diabetes and a previous history of gestational diabetes [210-213]. Women with gestational diabetes have a high risk of developing Type 2 diabetes later in life [214,215]. Fetal hyperglycaemia as an outcome of maternal hyperglycaemia can contribute to excessive fetal growth [202]. However, gestational diabetes has paradoxical effects of fetal growth with outcomes of increased or decreased birth weight [216]. About 85% of term newborn infants are born with birth weights in the normal range of 2500 to 4000 g. Among full-term infants, 7 to 8% of newborns have a birth weight of <2500 g (10 percentile) and a similar percentage are born overweight (>4000 g, 90 percentile) [216].

### Causes of excess cortisol levels during pregnancy

While cortisol levels rise as gestation progresses, there are events that may occur during the pregnancy that elevate cortisol further. Many of the listed prenatal risk factors for ASD have the potential to alter cortisol levels either directly through stimulating the adrenocortical cells, the HPA axis, or indirectly through modulating 11β-HSD1 expression. Conditions that increase cortisol production during gestation are listed below. For example, activation of cortisol production is important for a role in host response during acute or chronic stressful events such as sepsis and viral infections [217]. Also inflammatory cytokines, such as the interleukins (IL-1, IL-6) and TNF-α acting at the pituitary and adrenocortical levels, stimulate cortisol formation [217]. In patients with rheumatoid arthritis, the cytokines TNF-α, IL-6 and IL-1 cause inflammation of the synovial joints [218], which would alter cortisol production in this cohort. Adenovirus and cytomegalovirus have been shown to sequester into the adrenocortical cells to stimulate cortisol production [219,220]. Iron deficiency has been reported to elevate cortisol levels in pregnant women through increased synthesis of CRH, resulting in increased risk of preterm labour, hypertension and pre-eclampsia [221]. Additionally, haemoglobin levels are inversely correlated with IL-6 levels [222]. Uncontrolled asthma is associated with reduced placental 11β-HSD2 activity, which significantly increases fetal cortisol levels [223]. Glucocorticoids are now the consensus treatment for preterm labour occurring between gestational weeks 24 and 34 in nearly one in ten pregnancies in the USA, to prevent the adverse consequences of respiratory distress syndrome [224]. Prenatal depression and psychological stress are associated with elevated cortisol levels, prematurity and low birth weights [225,226]. Increased waist circumference associated with advancing age, even in the absence of weight gain [227] and obesity, are associated with increased cortisol production [228,229].

### Conditions during pregnancy that elevate cortisol levels

#### Condition

1. Obesity [229]

2. Infection [220]

3. Psychological stress [226]

4. Depression [225]

5. Asthma [223]

6. Iron deficiency [221]

7. Preterm labour^a^[224]

^a^Requiring dexamethasone treatment.

### Cortisol and the placenta

In mammals glucocorticoids are central to fetal growth, tissue development and maturation of various organs [230]. Supraphysiological levels of glucocorticoids cause fetal growth retardation in mammalian models and humans, and reduced intrauterine growth is associated with high maternal and fetal concentrations of glucocorticoids [231,232]. Glucocorticoids are lipophilic and readily cross the placenta, and in rat studies prenatal exposure to the synthetic glucocorticoids dexamethasone or betamethasone reduces birth weight [233]. Normally, fetal physiological glucocorticoid levels are lower than the maternal levels [234]. This gradient is achieved by fetoplacental 11β-HSD2, which metabolises cortisol [235]. This barrier is not impervious and as such a minor percentage of maternal cortisol crosses to the fetus [236]. The efficiency of placental 11β-HSD2 varies considerably [233,237] where the lowest placental 11β-HSD2 activity and presumably, highest fetal exposure to glucocorticoids, results in lower birth weights [223,237].

Some children diagnosed with ASD or who have higher scores on ASD spectrum screening have low birth weights [20,238,239], which could be an outcome of elevated cortisol levels that occur during the prenatal period. Furthermore, the placenta of female fetuses have increased glucocorticoid inactivation and lower corticoid receptor density than the placentas of males [240], which may render males more vulnerable to elevated maternal cortisol levels and explain gender differences in the prevalence of ASD.

### Cortisol and SERT expression

As excess cortisol is associated with many serotonin-derived pathological conditions, its role in altering serotonin function should be investigated. Certainly, evidence is emerging for an effect of cortisol on SERT expression in cells and tissue. Dexamethasone, a synthetic glucocorticoid, has been demonstrated to increase the mRNA and protein expression of SERT in immortalized human B-lymphoblastoid cells [241], an effect that was dependent on a region located 1.4 kb upstream of the 5HTT gene transcription site and was elevated in both polymorphisms of the 5-HTTLPR promoter sequence. Stress and elevated cortisol has been demonstrated to elevate tissue SERT expression in rodent and human studies [242-244]. In a rodent model, maternal administration of dexamethasone during gestation produced persistent increases in (3H^+^) paroxetine (SSRI) binding to SERT in the brainstem and cortex without affecting the numbers of serotonergic nerve terminals [245]. Interestingly, alterations in brain serotonin levels were not offset by changes in the fractional serotonin turnover rate [246].

Both forms of the 5-HTTLPR promoter sequence polymorphisms have been reported to be associated with ASD [113-118]. However, neither polymorphism in the promoter sequence has been demonstrated conclusively to be associated with ASD. Similarly, allelic variants within the 5-HTTLPR, have been reported, which indicates that variants within the S and L alleles occur. These variants have been investigated in a Caucasian and Japanese population, demonstrating significant ethnic differences for the distribution of alleles and genotypes [247]. However, common polymorphism/s in the 5-HTTLPR promoter sequence that confers increased subsceptibility to elevated cortisol levels in the GR, GRE or coactivator sites, should be considered as a focus of genetic studies. The GRE consensus sequence, the hexanucleotide TGTTCT, was originally proposed, however, further evidence has indicated that the GRE can involve an imperfect inverted repeat of the hexanucleotide TGTTCT with a 3-bp spacer to create a palindromic structure [248]. The GRE is very similar in nucleotide sequence to the oestrogen response element (ERE) where minor changes in base sequence for the ERE can be converted to a GRE [249,250]. Changes in the nucleotide sequence can also confer altered sensitivity to GR-induced function [249,250]. Furthermore, the GR interaction with the palindromic site promotes an allosteric change in the DNA-binding domain, to alter the protein shape and promote dimerization with a second GR monomer subunit [248]. Mutations in the GR DNA-binding domain (Ser459Ala) and D loop (Pro493Arg) causes the formation of a constitutive dimerization interface, inducing GR dimerization on a non-specific DNA [251]. Other SNPs in the GR, including ER22/23EK, N363S and Bc*II* have been found to be associated with glucocorticoid sensitivity and stress-induced cortisol responses, and provide potential targets for investigating the genetic inheritance of ASD [252].

### Evidence for elevated SERT or cortisol in children with ASD

It is the premise of this review that excessive plasma levels of cortisol during pregnancy increase the expression of the SERT transporter, to alter serotonin levels during gestation and modify prenatal neuronal development in children diagnosed with ASD. Hence the best time to measure changes in SERT expression would be immediately after birth. During infancy the SERT expression will alter according to cortisol levels. Due to the limitations of diagnosis in this cohort, most research is completed in children age 4 to 9 years or older, and as such, work to investigate neuronal SERT expression is too late, as protein levels would be highly variable. Subsequently, the therapeutic use of SSRIs in children with ASD has been questioned as this class of drug has been demonstrated to have limited use in this cohort. Again it would be anticipated that SSRI use would be helpful when SERT expression is highest in younger children [253]. Elevated platelet serotonin levels (or SERT mRNA) may provide a simple test of elevated SERT expression in newborn children.

Alternatively, we could appraise evidence of exposure to excess cortisol in newborn children; however, again there is little research that has been done in neonates. Reviewing the effects of extraphysiological cortisol (see Table 1), the following symptoms in newborn children could be expected: insomnia, irritability, low cortisol levels or low bone density. Two studies have measured bone density in boys diagnosed with ASD (age 4 to 14 years) and found reductions in bone mineral density or bone cortical thickness. The authors attributed these findings to low vitamin D levels or diet [254,255]. These findings did not account for the possibility that they may have started from a lower base. Also, children who are subjected to high cortisol levels in utero are at risk of developing diabetes, however little research has been reported on the long-term physical outcomes of adults with ASD [256].

## Conclusions

This review investigates the potential causes of ASD and has focused on disruptions in serotonin signalling caused by elevated SERT levels during the prenatal period. Increases in membrane SERT levels are driven in utero by elevated cortisol levels. Increased cortisol in pregnant women may result in various clinical presentations including gestational diabetes, hypertension or depression. It may also be caused by excess anxiety or stress and inflammatory disorders such as asthma or infections. Further studies of excessive free cortisol levels during pregnancy should be done in women who have previously had children with ASD, or have a family history of anxiety, depression or diabetes. A standard protocol to measure free-cortisol levels in women during gestation needs to be established. Methods to measure free-cortisol levels include calculated free cortisol (Coolen’s method), calculating the free-cortisol index (ratio of serum cortisol to transcortin concentrations) or quantifying salivary cortisol [217]. Cortisol is vital for fetal development with respect to maturation of lung function; however, prenatal exposure to excess glucocorticoids has also been shown to be detrimental to fetal growth and have longer-term effects related to hypertension and type II diabetes in adult life [257,258].

## Abbreviations

ACTH: Adrenocorticotrophic hormone; ASD: Autism spectrum disorder; CBG: Corticosteroid binding globulin; CBP: Corticosteroid binding protein; CRH: Corticotrophin releasing hormone; CSF: Cerebral spinal fluid; DAT: Dopamine transporter; ERE: Estrogen response element; G6Pase: Glucose-6-phosphatase; GR: Glucocorticoid receptor; GRE: Glucocorticoid response elements; 5-HIAA: 5-hydroxyindolacetic; 11β-HSD: 11β-hydroxysteroid dehydrogenase; 5-HT: 5-hydroxytryptamine; 5-HTTP: 5-hydroxytryptophan; 5-HTTLP: Serotonin transporter gene promoter region; HPA: Human hypothalamic axis; IFN-γ: Interferon-γ; IL: Interleukin; MAPK: Mitogen-activated protein kinase; NADP(H): Nicotinamide adenine dinucleotide phosphate reduced; NET: Noradrenaline transporter; PDD-NOS: Pervasive developmental disorder not otherwise specified; PET: Positron emission tomograhy; PEPCK-C: Phosphoenolpyruvate carboxykinase; PK: Protein kinase; SERT: Serotonin reuptake transporter; SNP: Single nucleotide polymorphism; SSRI: Selective serotonin reuptake inhibitor; SLC6A4: Solute carrier family 6 (neurotransmitter transporter, serotonin), member 4; TNF-α: Tumour necrosis factor-α; VNT: Variable number of tandem repeats.

## Competing interests

The author has no competing interest.
